# Course of Encephalopathy in a Cirrhotic Dialysis Patient Treated Sequentially with Peritoneal and Hemodialysis

**DOI:** 10.1155/2015/375456

**Published:** 2015-03-25

**Authors:** Suleyman Koz, Idris Sahin, Zafer Terzi, Sema Tulay Koz

**Affiliations:** ^1^Nephrology Clinic, Malatya State Hospital, 44100 Malatya, Turkey; ^2^Nephrology Department, Inonu University Turgut Ozal Medical Centre, 44100 Malatya, Turkey; ^3^Internal Medicine Clinic, Adiyaman State Hospital, Adiyaman, Turkey; ^4^Dialysis Clinic, Malatya State Hospital, 44100 Malatya, Turkey

## Abstract

End-stage kidney disease and advanced cirrhosis are sometimes seen concomitantly. There is no consensus on dialysis modality in terms of determining the optimal way of treating these patients. It has been suggested that peritoneal dialysis is a better choice for these patients, but efficacy of hemodialysis in stable cirrhotic patients has not been evaluated sufficiently. We report a case with advanced cirrhosis and end-stage kidney disease who was faced with hepatic encephalopathy episodes up on starting renal replacement therapy. The case is also interesting in that it reveals effects of hemodialysis and peritoneal dialysis on hepatic encephalopathy episodes and quality of life of the patient.

## 1. Introduction

Advanced cirrhosis and end stage kidney diseases (ESKD) are sometimes seen concomitantly. There is no consensus on optimal renal replacement treatment (RRT) that should be offered to these patients. Here we report our experience in an advanced stage cirrhotic patient with ESKD in whom we performed peritoneal dialysis (PD) and hemodialysis (HD) sequentially.

## 2. The Case

Thirty-year old male patient diagnosed as advanced stage cryptogenic cirrhosis (Child-Pugh classification category C) was admitted to our clinic because of ESKD. A Tenckhoff catheter was implanted blindly to peritoneum for RRT. PD was chosen because of preference of the patient as well as opportunity of treatment of ascites through it. Prior to the catheter implantation approximately 15 mL/kg fresh frozen plasma was infused. Of this 10 mL/kg was infused 12 hours and 5 mL/kg was infused just 2 hours prior to the procedure. There was no need to fill the abdomen with dialysis solution prior to the procedure, because the patient already had massive ascites. Other steps of the procedure were the same as we practice in our clinic. After the catheter placement we drained 1000 mL of ascites and terminated the procedure. There were no serious complications related to catheter implantation. Ecchymosis and a subcutaneous hematoma of about 3 cm diameter were seen, and both of them resolved spontaneously. The catheter has been flushed every other day and, each time, 500 to 700 mL of ascites has been drained. There were no mental changes and hemodynamic changes at this period. Routine dialysis changes started at the day of 21.

Number and volume of dialysis changes were gradually increased to four changes per day. We have used 2000 cc, 1.36% glucose containing solutions with electrolyte concentration of Na 132 mmol/L, Ca 1.25 mmol/L, and Mg 0.25 mmol/L. Daily fluid intake and output of the patient were approximately equal. There were no episodes of hepatic encephalopathy (HE) until the patient started to perform 3 to 4 exchanges daily. Thereafter, frequently, the patient was faced with somnolence, stupor, and coma (grades 3 and 4 HE according to West Haven scoring system [1]) without an obvious precipitating factor. Later on we came to conclusion that episodes of encephalopathy could be related to PD. Then we decided to hold PD temporarily and shift the patient to HD. Upon shifting to HD, encephalopathy episodes were dramatically reduced. Dialysate flow rate was set to 500 mL/min in all sessions, and its electrolyte concentrations were as follows: Na 140 mmol/L, Ca 1.25 mmol/L, Mg 0.5 mmol/L, and K 2 mmol/L. Blood flow rate was a round 300 mL/min throughout the sessions. Mean duration of the session length was 160 minute (min–max: 120–180 min); mean ultrafiltration volume was 504 mL per session (min–max: 0–1900 cc). Mean of mean arterial pressure (MAP) measurements at the beginning of the sessions and mean of lowest MAP measurements during the sessions were 121.4 and 115.0 mmHg, respectively. After the period of HD, PD was tried once more, and the patient, again, was faced with frequent grades 3 and 4 HE episodes. Then the patient was shifted to HD permanently almost free of HD related acute complications, such as bleeding and hemodynamic instability. The patient was faced with two episodes of peritonitis during the entire period of PD, with one being just after the day of catheter implantation. Cultures have grown no microorganism, and the patient was treated empirically with an antibiotic regimen routinely used to treat peritonitis in our clinic.

**Figure 1:**
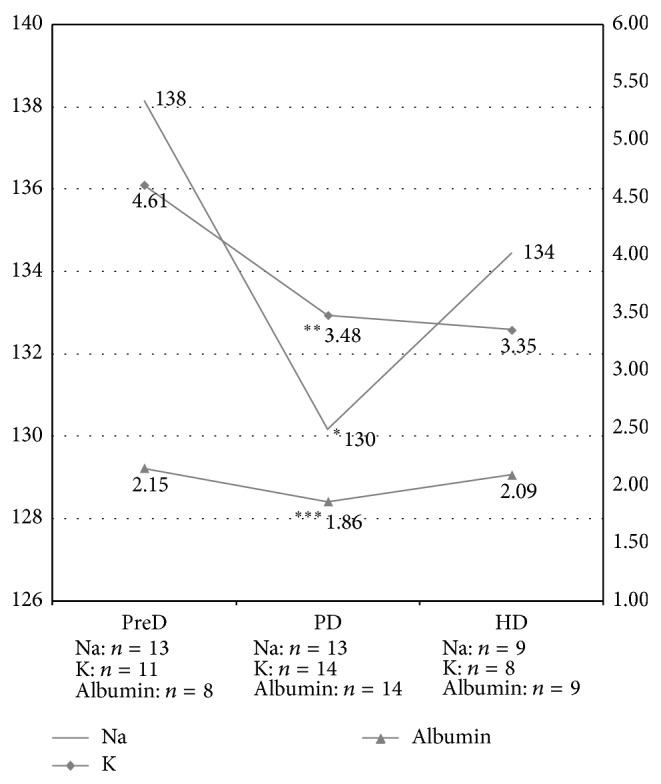
Mean Na, K, and albumin levels of the patient during PreD, PD and HD periods. The numbers on the graph lines represent the means. ^*^Na: Na level of PD is significantly lower than that of PreD, *P* = 0.004. Differences between other groups are not significant. ^**^K: K level of PD is significantly lower than that of PreD *P* = 0.006, and K level of HD is significantly lower than that of PreD *P* = 0.01. The difference between mean K level of PD and HD periods is not significant. ^***^Albumin: differences between groups are not statistically significant. *n*: number of laboratory values in the relevant period, PreD: predialysis period, PD: peritoneal dialysis period, and HD: hemodialysis period. Comparisons between groups were done by using Wilcoxon Signed Rank Test (Statistical Package designed for the Social Sciences (SPSS) software, version 17.0 (SPSS Inc., Chicago, IL, USA)), and *P* < 0.05 was considered as statistically significant.

Figure 1 summarizes data concerning serum Na, K, and albumin levels of the patient during the different periods of follow-up. The data were obtained, retrospectively, from electronic database of the hospital; all of the values present in the database were included in the analysis. In brief, mean Na and K levels were significantly lower during PD compared to predialysis (PreD) period. Six of the 13 Na measurements during the PD period were equal to or lower than 130 mmol/L. Analysis revealed no statistically significant differences between PD and HD in terms of mean Na, K, and albumin levels. Mean ammonia levels during PD and HD were 182.6 and 184.6 mg/dL, respectively. Mean blood urea nitrogen (BUN) levels during PD and HD periods were 96.5 ± 17.2 and 49.8 ± 27.5 (*P* < 0.05, Mann-Whitney *U* Test). Mean creatinine levels during PD and HD periods were 6.9 ± 1.4 and 4.7 ± 0.7 (*P* < 0.05, Mann-Whitney *U* Test).

## 3. Discussion

Medical reports on application of PD in ESKD patients with advanced chronic liver diseases mainly consist of case reports and small series [2–7]. It has been claimed that these patients cannot tolerate well HD because of hypotension and bleeding diathesis [5, 6, 8, 9]. On the other hand hemodynamic stability, lack of need for anticoagulation, and drainage of ascitic fluid are regarded as advantages of PD [2, 5, 9].

It is argued that PD allows relatively steady changes in fluid, solute, and toxic substances compared to HD; hence, it preserves delicate balance of the liver patients and prevents HE [3]. On the other hand, intermittent HD may result in increase in brain water content and intracranial pressure during the sessions [10–12]. It has also been suggested that intermittent HD may render patients in a vulnerable state for HE due to intradialytic hemodynamic and metabolic changes [8, 10].

In this case, in contrast to the expectations, we observed deterioration of mental status upon starting peritoneal dialysis. To tell openly, we do not have a clear explanation for this interesting situation. So, we can just speculate about it. Serum albumin, electrolyte, and osmotic changes may be culprit in deterioration of the patient's consciousness. It is stated that symptoms of hyponatremic encephalopathy are due to brain edema, and the symptoms begin at serum Na level of less than 130 mmol/L [9]. HE is a neuropsychiatric manifestation of the chronic liver disease and, similar to hyponatremic encephalopathy, characterized by brain edema and astrocyte swelling in addition to alterations in neurotransmitter balance and glucose metabolism. Electrolyte imbalances may incite HE [1]. Here we cannot distinguish HE from hyponatremic encephalopathy. Both hyponatremia and other factors might have had additive effects in development of mental alterations in the patient. As a matter of fact, hyponatremia, in cirrhotic patients, results in depletion of intracellular organic osmolytes and low-grade cerebral edema; it is a risk factor and predictive of HE [13]. Serum Na level is not expected to be changed in “normal” CAPD patients using solutions with Na concentration of around 130 mmol/L [14]. Again, it can be speculated that peritoneal ultrastructure might have been changed in cirrhosis, similar to the patients recovering from peritonitis, which might have resulted in altered Na transport. Another factor that might affect distribution of the body fluids and osmotic pressure is hypoalbuminemia; decreased plasma oncotic pressure might have resulted in increased intercellular fluid, decreased Na concentration, and decreased osmotic pressure. There are some indirect evidences supporting our view. Hypertonic sodium chloride (NaCl) decreases brain edema when infused to liver disease patients [15]. In acute neurological states higher sodium level of dialysate decreases complications of dialysis [16]. Relatively lower K level in the PD period, by increasing ammonia production, might be an additional factor in the genesis of HE [13]. Although BUN and creatinine levels were significantly higher during the PD period, we do not think that they have a significant role in development of the clinical picture, because their levels were higher during the predialysis period when the patient did not have encephalopathy episodes at all.

PD and hypoalbuminemia might change serum amino acid concentrations [17, 18]. It can be speculated that these changes might alter the neurotransmitter composition of brain. Consistently, a report suggested that serum amino acid composition might have a role in HE [19].

Quality of life of the patient, although not assessed formally, was markedly better than PD period; at least the patient could be discharged from the hospital.

In a previously reported case the situation was in contrast to the present case. The patient recovered from HE upon the shift from HD to PD. Again, the exact cause of the incident could not be clarified [20]. There is another case with HE during PD, and the encephalopathy improved partially upon the shift of the patient to HD. Later on, they had come to conviction that HE was due to spontaneous portosystemic shunting of blood, and subsequently, surgical closure of the shunt resulted in complete recovery from HE [21].

As a result, cirrhotic patients can be treated either with PD or HD. We suggest a flexible approach and individualized decisions according to patients' characteristics and clinical course.
